# Random KNN feature selection - a fast and stable alternative to Random Forests

**DOI:** 10.1186/1471-2105-12-450

**Published:** 2011-11-18

**Authors:** Shengqiao Li, E James Harner, Donald A Adjeroh

**Affiliations:** 1 The Department of Statistics, West Virginia University, Morgantown, WV 26506, USA; 2 Health Effects Laboratory Division, the National Institute for Occupational Safety and Health, Morgantown, WV 26505, USA; 3 The Lane Department of Computer Science and Electrical Engineering, West Virginia University, Morgantown, WV 26506, USA

## Abstract

**Background:**

Successfully modeling high-dimensional data involving thousands of variables is challenging. This is especially true for gene expression profiling experiments, given the large number of genes involved and the small number of samples available. Random Forests (RF) is a popular and widely used approach to feature selection for such "small *n*, large *p *problems." However, Random Forests suffers from instability, especially in the presence of noisy and/or unbalanced inputs.

**Results:**

We present RKNN-FS, an innovative feature selection procedure for "small *n*, large *p *problems." RKNN-FS is based on Random KNN (RKNN), a novel generalization of traditional nearest-neighbor modeling. RKNN consists of an ensemble of base *k*-nearest neighbor models, each constructed from a random subset of the input variables. To rank the importance of the variables, we define a criterion on the RKNN framework, using the notion of *support*. A two-stage backward model selection method is then developed based on this criterion. Empirical results on microarray data sets with thousands of variables and relatively few samples show that RKNN-FS is an effective feature selection approach for high-dimensional data. RKNN is similar to Random Forests in terms of classification accuracy without feature selection. However, RKNN provides much better classification accuracy than RF when each method incorporates a feature-selection step. Our results show that RKNN is significantly more stable and more robust than Random Forests for feature selection when the input data are noisy and/or unbalanced. Further, RKNN-FS is much faster than the Random Forests feature selection method (RF-FS), especially for large scale problems, involving thousands of variables and multiple classes.

**Conclusions:**

Given the superiority of Random KNN in classification performance when compared with Random Forests, RKNN-FS's simplicity and ease of implementation, and its superiority in speed and stability, we propose RKNN-FS as a faster and more stable alternative to Random Forests in classification problems involving feature selection for high-dimensional datasets.

## Background

Selection of a subset of important features (variables) is crucial for modeling high dimensional data in bioinformatics. For example, microarray gene expression data may include *p *≥ 10, 000 genes. But the sample size, *n*, is much smaller, often less than 100. A model cannot be built directly since the model complexity is larger than the sample size. Technically, linear discriminant analysis can only fit a linear model up to *n *parameters. Such a model would provide a perfect fit, but it has no predictive power. This "*small n, large p *problem" has attracted a lot of research attention, aimed at removing nonessential or noisy features from the data, and thus determining a relatively small number of features which can mostly explain the observed data and the related biological processes.

Though much work has been done, feature selection still remains an active research area. The significant interest is attributed to its many benefits. As enumerated in [1], these include (i) reducing the complexity of computation for prediction; (ii) removing information redundancy (cost savings); (iii) avoiding the issue of overfitting; and (iv) easing interpretation. In general, the generalization error becomes lower as fewer features are included, and the higher the number of samples per feature, the better. This is sometimes referred to as the *Occam's razor *principle [2]. Here we give a brief summary on feature selection. For a recent review, see [3]. Basically, feature selection techniques can be grouped into three classes: *Class I*: *Internal variable selection*. This class mainly consists of *Decision Trees *(DT) [4], in which a variable is selected and split at each node by maximizing the purity of its descendant nodes. The variable selection process is done in the tree building process. The decision tree has the advantage of being easy to interpret, but it suffers from the instability of its hierarchical structures. Errors from ancestors pass to multiple descendant nodes and thus have an inflated effect. Even worse, a minor change in the root may change the tree structure significantly. An improved method based on decision trees is Random Forests [5], which grows a collection of trees by bootstrapping the samples and using a random selection of the variables. This approach decreases the prediction variance of a single tree. However, Random Forests may not remove certain variables, as they may appear in multiple trees. But Random Forests also provides a variable ranking mechanism that can be used to select important variables.

*Class II: Variable filtering*. This class encompasses a variety of filters that are principally used for the classification problem. A specific type of model may not be invoked in the filtering process. A filter is a statistic defined on a random variable over multiple populations. With the choice of a threshold, some variables can be removed. Such filters include *t*-statistics, *F*-statistics, Kullback-Leibler divergence, Fisher's discriminant ratio, mutual information [6], information-theoretic networks [7], maximum entropy [8], maximum information compression index [9], relief [10,11], correlation-based filters [12,13], relevance and redundancy analysis [14], etc.

*Class III: Wrapped methods*. These techniques wrap a model into a search algorithm [15,16]. This class includes foreward/backword, stepwise selection using a defined criterion, for instance, partial *F*-statistics, Aikaike's Information Criterion (AIC) [17], Bayesian Information Criterion (BIC) [18], etc. In [19], sequential projection pursuit (SPP) was combined with partial least square (PLS) analysis for variable selection. Wrapped feature selection based on Random Forests has also been studied [20,21]. There are two measures of importance for the variables with Random Forests, namely, mean decrease accuracy (MDA) and mean decrease Gini (MDG). Both measures are, however, biased [22]. One study shows that MDG is more robust than MDA [23]; however another study shows the contrary [24]. Our experiments show that both methods give very similar results. In this paper we present results only for MDA. The software package **varSelRF **in R developed in [21] will be used in this paper for comparisons. We call this method RF-FS or RF when there is no confusion. Given the hierarchical structure of the trees in the forest, stability is still a problem.

The advantage of the filter approaches is that they are simple to compute and very fast. They are good for pre-screening, rather than building the final model. Conversely, wrapped methods are suitable for building the final model, but are generally slower.

Recently, *Random KNN *(RKNN) which is specially designed for classification in high dimensional datasets was introduced in [25]. RKNN is a generalization of the *k*-nearest neighbor (KNN) algorithm [26-28]. Therefore, RKNN enjoys the many advantages of KNN. In particular, KNN is a nonparametric classification method. It does not assume any parametric form for the distribution of measured random variables. Due to the flexibility of the nonparametric model, it is usually a good classifier for many situations in which the joint distribution is unknown, or hard to model parametrically. This is especially the case for high dimensional datasets. Another important advantage of KNN is that missing values can be easily imputed [29,30]. Troyanskaya *et al*. [30] also showed that KNN is generally more robust and more sensitive compared with other popular classifiers. In [25] it was shown that RKNN leads to a significant performance improvement in terms of both computational complexity and classification accuracy. In this paper, we present a novel feature selection method, RKNN-FS, using the new classification and regression method, RKNN. Our empirical comparison with the Random Forests approach shows that RKNN-FS is a promising approach to feature selection for high dimensional data.

## Methods

### Random KNN

The idea of Random KNN is motivated by the technique of Random Forests, and is similar in spirit to the method of random subspace selection used for Decision Forests [31]. Both Random Forests and Decision Forests [31] use decision trees as the base classifiers. Compared with the two, Random KNN uses KNN as base classifiers, with no hierarchical structure involved. Compared with decision trees, KNN is simple to implement and is stable [32]. Thus, Random KNN can be stabilized with a small number of base KNN's and hence only a small number of important variables will be needed. This implies that the final model with Random KNN will be simpler than that with Random Forests or Decision Forests. Specifically, a collection of *r *different KNN classifiers will be generated. Each one takes a random subset of the input variables. Since KNN is stable, bootstrapping is not necessary for KNN. Each KNN classifier classifies a test point by its majority, or weighted majority class, of its *k *nearest neighbors. The final classification in each case is determined by majority voting of *r *KNN classifications. This can be viewed as a sort of voting by a *Majority of a Majority.*

More formally, let **F **= {*f*_1_, *f*_2_,..., *f*_*p*_} be the *p *input features, and **X **be the *n *original input data vectors of length *p*, i.e., an *n *× *p *matrix. For a given integer *m *<*p*, denote **F**^(*m*) ^= {*f*_*j*1_, *f*_*j*2_,..., *f*_*jm *_|*f*_*jl *_∈ **F**, 1 ≤ *l *≤ *m*} a random subset drawn from **F **with equiprobability.

Similarly, let **X**^(*m*) ^be the data vectors in the subspace defined by **F**^(*m*)^, i.e., an *n *× *m *matrix. Then a **KNN**^(*m*) ^classifier is constructed by applying the basic KNN algorithm to the random collection of features in **X**^(*m*)^. A collection of *r *such base classifiers is then combined to build the final Random KNN classifier.

### Feature support - a ranking criterion

**Figure 1:**
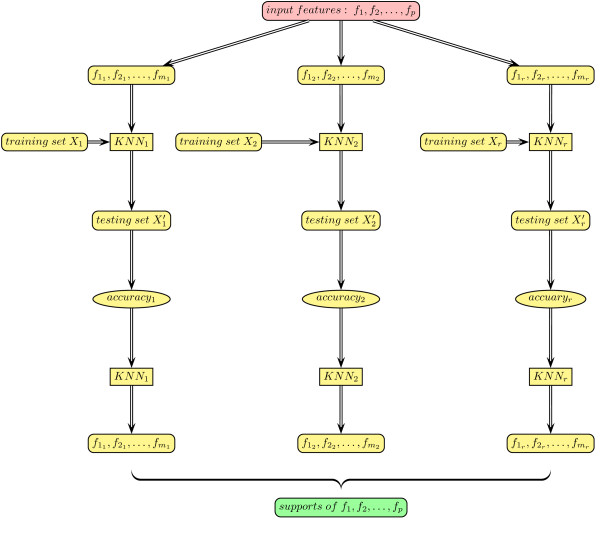
**Bidirectional voting using Random KNN**.

In order to select a subset of variables that have classification capability, the key is to define some criteria to rank the variables. We define a measure, called *support*. Each feature *f *will appear in some KNN classifiers, say, set **C**(*f*) of size *M*, where *M *is the multiplicity of *f*. In turn, each classifier *c *∈ **C**(*f*) is an evaluator of its *m *features, say, set **F**(*c*). We can take its accuracy as a performance measure for those features. The mean accuracy of these KNN classifiers (support) is a measure of the feature relevance with the outcome. Thus we have a ranking of the features. We call this scheme *bidirectional voting*. Each feature randomly participates in a series of KNNs to cast a vote for classification. In turn, each classification result casts a vote for each participating feature. The algorithm is listed in Table 1. A schematic diagram of the bidirectional voting procedure is shown in Figure 1.

**Table 1:** Computing feature supports using Random KNN bidirectional voting

/* Generate *n *KNN classifiers using *m *features and compute accuracy *acc *for each KNN */
/* Return support for each feature */
*p *← number of features in the data set;
*m *← number of features for each KNN;
*r *← number of KNN classifiers;
*F*_*i *_← feature list for *i*^*th *^KNN classifier;
*C *← build *r *KNNs using *m *feature for each;
Perform query from base data sets using each KNN;
Compare predicted values with observed values;
Calculate accuracy, *acc*, for each base KNN;
$F\leftarrow {⋃}_{i=1}^{r}F_{i}$; {*F *is the list of features that appeared in *r *KNN classifiers};
**for **each *f *∈ *F ***do**
*C*(*f*) ← list of KNN classifiers that used *f*;
$support\left(f\right)\leftarrow \frac{1}{\left|C\left(f\right)\right|}\sum_{knn\in C\left(f\right)}acc\left(knn\right);$
**end for**

To compute feature supports, data are partitioned into base and query subsets. Two partition methods may be used: (1) dynamic partition: For each KNN, the cases are randomly partitioned. One half is the base subset and the other half is the query subset; (2) the data set is partitioned once, and for all KNN's, the same base subset and query subset are used. That is, all base subsets are the same and all query subsets are also the same. For diversity of KNN's, the dynamic partition is preferred.

**Figure 2:**
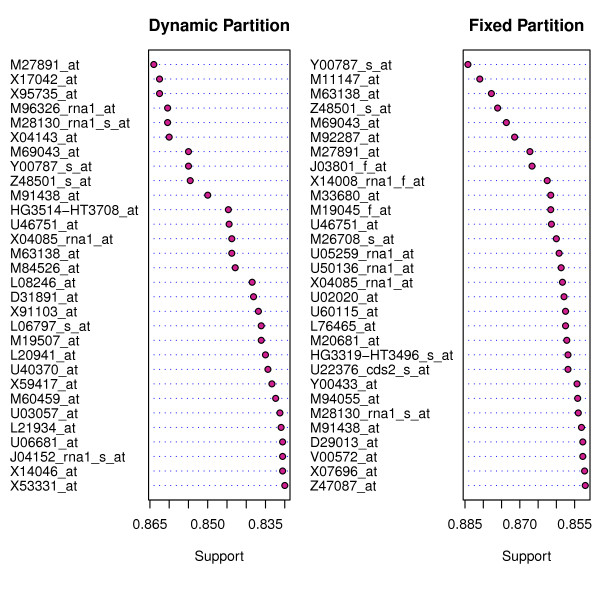
**Supports for the first 30 most relevant genes using the Golub leukemia data (Left panel: using dynamic partition; Right panel using fixed partition of the data for testing and training)**.

*Support *is an importance measure. The higher the support, the more relevant the feature. Figure 2 shows the 30 most relevant genes determined using the support criterion for Golub's 38 leukemia training samples, for both fixed and dynamic partitions. The dataset is available in an R package **golubEsets**.

### RKNN feature selection algorithm

With feature supports, we can directly select high rank features after running the support algorithm on the entire data set. We call this *direct selection*. But this simple approach may be too aggressive and risky for high dimensional data. We take a more conservative and safer approach, namely, multiple rounds of screening. That is, we recursively apply the direct selection procedure. To balance between speed and classification performance, we split recursion into two stages. The first stage is fast, and the number of variables is reduced by a given ratio (1/2 by default). This stage is a geometric elimination process since the dimension to be kept is a geometric progression. In the second stage, a fixed number of features (one by default) are dropped each time. This is a linear reduction process. Finally, a relatively small set of variables will be selected for the final models. To aid in this recursive procedure, another assessment criterion for a set of features is required. We use the average accuracy of the *r *random KNNs. After the first stage, we can plot the average accuracies against the number of features. The iteration just before the maximum accuracy is reached is called *pre-max *iteration. The feature set from the pre-max iteration will be the input for the second stage selection. The algorithm is shown in Table 2.

**Table 2:** Two-stage variable backward elimination procedure for Random KNN

**Figure 3:**
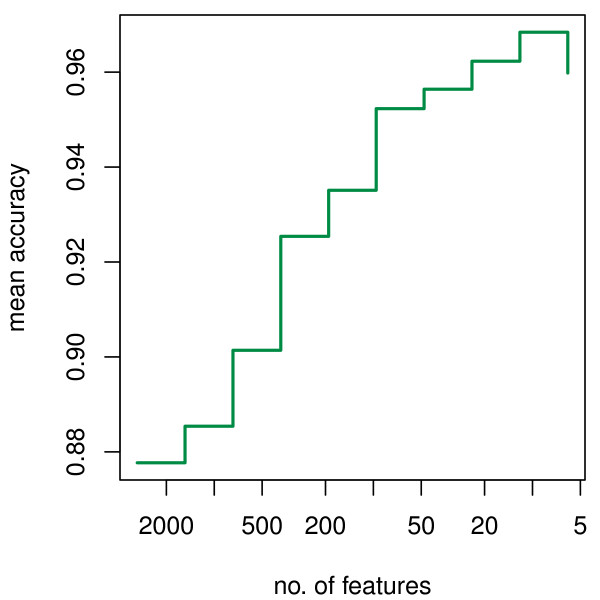
**Mean accuracy change with the number of features for the Golub leukemia data in the first stage**.

**Figure 4:**
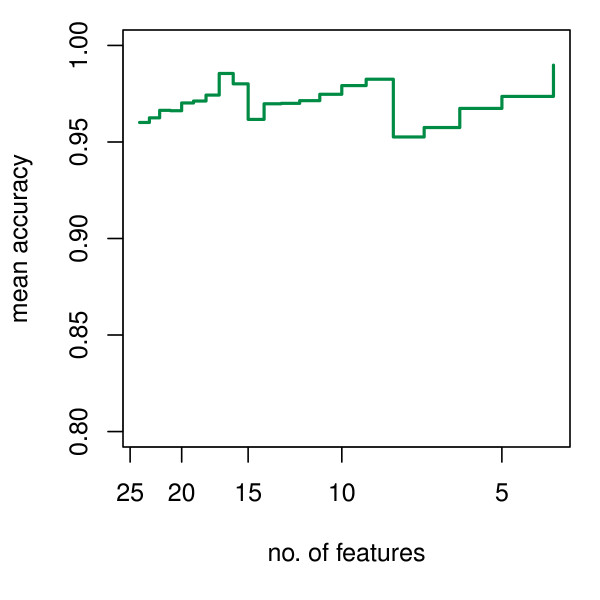
**Mean accuracy change with the number of features for the Golub leukemia data in the second stage (feature set with peak value is selected)**.

Stage 1: Geometric Elimination
*q *← proportion of the number features to be dropped each time;
*p *← number of features in data;
$ni\leftarrow \left\lfloor \ln\left(4∕p\right)∕\ln\left(1-q\right)\right\rfloor$; /* number of iterations, minimum dimension 4*/
initialize *rknn*_*list*[*m*]; /* stores feature supports for each Random KNN */
initialize *acc*[*m*]; /* stores accuracy for each Random KNN */
**for ***i *from 1 to *ni ***do**
if *i *== 1 **then**
*rknn *← compute supports via Random KNN from all variables of data;
**else**
$p\leftarrow \left\lfloor p\cdot \left(1-q\right)\right\rfloor ;$
*rknn *← compute supports via Random KNN from *p *top important variables of *rknn*;
**end if**
*rknn list*[*i*] ← *rknn;*
*acc*[*i*] ← accuracy of *rknn*;
**end for**
$max=\underset{1\leq k\leq ni}{\mathrm{argmax}}\left(acc\left[k\right]\right);$
*pre*_*max *= *max *- 1;
*rknn *← *knn*_*list*[*pre*_*max*]; /* This Random KNN goes to stage 2 */
Stage 2: Linear Reduction
*d *← number features to be dropped each time;
*p *← number of variables of *rknn*;
$ni\leftarrow \left\lfloor \left(p-4\right)∕d\right\rfloor$; /* number of iterations */
**for ***i *from 1 to *ni ***do**
**if ***i *≠ 1 **then**
*p *← *p *- *d*;
**end if**
*rknn *← compute supports via Random KNN from *p *top important variables of *rknn*;
*acc*[*i*] ← accuracy of *rknn*;
*rknn_list*[*i*] ←*rknn;*
**end for**
$best\leftarrow \underset{1\leq k\leq ni}{\mathrm{argmax}}\left(acc\left[k\right]\right);$
*best*_*rknn *← *rknn*_*list*[*best*]; /* This gives final random KNN model */
**return ***best*_*rknn*;

This procedure was applied to Golub's leukemia datasets. Figure 3 shows the variation of mean accuracy with decreasing number of features in the first stage of feature selection. Figure 4 shows the variation of mean accuracy with decreasing number of features in the second stage. From Figure 4, a maximum mean accuracy is reached when 4 genes are left in the model. These final four genes selected for leukemia classification are: X95735_at, U27460_at, M27891_at and L09209_s_at. Using these four genes and the ordinary KNN classifier (*k *= 3) to classify the 34 independent test samples, 18 of 20 ALL cases are correctly classified and 13 of 14 AML cases are correctly classified. Total accuracy is 91%. This model is very simple compared with others that use far more genes.

### Time complexity

#### Time complexity for computing feature support

For each KNN, we have the typical time complexity as follows:

• Data Partition: *O*(*n*);

• Nearest Neighbor Searching: *O*(*k*2^*m*^*n *log *n*);

• Classification: *O*(*kn*);

• Computing accuracy: *O*(*n*).

Adding the above 4 items together, we get a time needed for one KNN: *O*(*k*2^*m*^*n *log *n*). For Random KNN, we have *r *KNN's; thus the total time for the above steps is *O*(*rk*2^*m*^*n *log *n*). Since *rm *features are used in the Random KNN, the time for computing supports from these accuracies is *O*(*rm*). Thus the overall time is *O*(*rk*2^*m*^*n *log *n*) + *O*(*rm*) = *O*(*r*(*m *+ *k*2^*m*^*n *log *n*)) = *O*(*rk*2^*m*^*n *log *n*). Sorting these supports will take *O*(*p *log *p*). Since for most applications, log *p *<*n *log *n*, and *p *<*rk*2^*m*^, the time complexity for computing and ranking feature supports still remains as *O*(*rk*2^*m*^*n *log *n*).

#### Time complexity for feature selection

In stage-one, the number of features decreases geometrically with proportion *q*. For simplicity, let us take *m *to be the square-root of *p *and keep *r *fixed. Thus the sum of the component 2^*m *^is $2^{\sqrt{p}}+2^{\sqrt{pq}}+2^{\sqrt{pq^{2}}}+2^{\sqrt{pq^{3}}}+2^{\sqrt{pq^{4}}}+....$ The first term is dominant, since *q *is a fraction. Thus the time complexity will be in $O\left(rk2^{\sqrt{p}}n\log n\right)$.

In stage-two, each time a fixed number of features is removed. In the extreme case, only one feature is removed per iteration, the total time will be $O\left(rk2^{p_{1}+1}n\log n\right)$, where *p*_1 _is the number of features at the start of stage-two, and usually *p*_1 _< p^1/2^. So on average, we have time in $O\left(rk2^{p_{1}+1}n\log n\right)=O\left(rk2^{\sqrt{p}}n\log n\right)$.

Therefore, the total time for the entire algorithm will be in $O\left(rk2^{\sqrt{p}}n\log n\right)$, the same as that for using Random KNN for classification, at $m=\sqrt{p}$ Basically, in theory, feature selection does not degrade the complexity of Random KNN. With *m *= log *p*, we obtain time complexity in *O*(*rkpn *log *n*). This is significant, as it means that with appropriate choice of *m*, we can essentially turn the exponential time complexity of feature selection to linear time, with respect to *p*, the number of variables.

### Parameter setting

**Figure 5:**
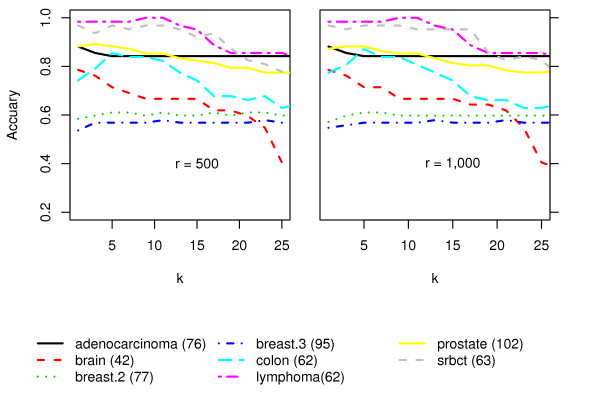
**The effect of *k***.

**Figure 6:**
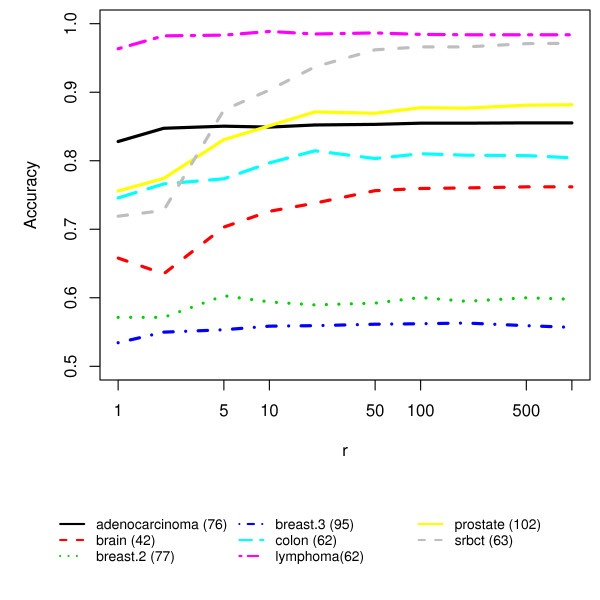
**The effect of *r*, the number of KNN base classifiers**.

The Random KNN has three parameters, the number of nearest neighbors, *k*; the number of random KNNs, *r*; and the number of features for each base KNN, *m*. For "small *n*, large *p*" datasets, *k *should be small, such as 1 or 3, etc. (see Figure 5), since the similarities among data points are related to the nearness among them. For *m*, we recommend $m=\sqrt{p}$ in order to maximize the difference between feature subsets [25]. Performance generally improves with increasing *r*, however, beyond a point, larger values of *r *may not lead to much further improvements. (See Figure 6 for experimental results). Beyond *r *> 1000, there is not much added advantage with respect to classification accuracy.

## Results and discussion

### Microarray datasets

To evaluate the performance of the proposed RKNN-FS, we performed experiments on 21 microarray gene expression datasets (Table 3 and 4). Ten of them were previously used to test the performance of Random Forests in gene selection [21]. These are available at http://ligarto.org/rdiaz/Papers/rfVS/randomForestVarSel.html. The other eleven were downloaded from http://www.gems-system.org. Some datasets are from the same studies but used different preprocessing routines, and thus the dimensionalities are different. These datasets are for gene profiling of various human cancers. The number of genes range from 2,000 to 15,009. The number of classes range from 2 to 26.

**Table 3:** Microarray gene expression datasets, Group I

Dataset	Sample Size, *n*	No. of Genes, *p*	No. of classes, *c*	*p*/*n*	p * *c*/*n*
Ramaswamy	308	15009	26	49	1267
Staunton	60	5726	9	95	859
Nutt	50	10367	4	207	829
Su	174	12533	11	72	792
NCI60	61	5244	8	86	688
Brain	42	5597	5	133	666
Armstrong	72	11225	3	156	468
Pomeroy	90	5920	5	66	329
Bhattacharjee	203	12600	5	62	310
Adenocarcinoma	76	9868	2	130	260
Golub	72	5327	3	74	222
Singh	102	10509	2	103	206

**Table 4:** Microarray gene expression datasets, Group II

Dataset	Sample Size, *n*	No. of Genes, *p*	No. of classes, *c*	*p*/*n*	p * *c*/*n*
Lymphoma	62	4026	3	65	195
Leukemia	38	3051	2	80	161
Breast.3.Classes	95	4869	3	51	154
SRBCT	63	2308	4	37	147
Shipp	77	5469	2	71	142
Breast.2.Classes	77	4869	2	63	126
Prostate	102	6033	2	59	118
Khan	83	2308	4	28	111
Colon	62	2000	2	32	65

Classwise sample sizes are from 2 to 139 (i.e., some datasets are unbalanced). The ratio of the number of genes, *p*, to the sample size, *n*, reflects the difficulty of a dataset and is listed in the table. The number of classes *c*, has a similar effect on the classification problem. Thus collectively, the quantity (*p*/*n*) * *c *is included in the tables as another measure of complexity of the classification problem for each dataset. Based on this, we divided the datasets into two groups - **Group I **- those with relatively high values for (*p*/*n*) * *c *(corresponding to relatively more complex classification problems), and **Group II **- those with relatively low values (corresponding to the datasets that present relatively simpler classification problems). We have organized our results around this grouping scheme.

### Evaluation methods

In this study, we compare Random KNN with Random Forests since they both are ensemble methods. The difference is the base classifier. We perform leave-one-out cross-validation (LOOCV) to obtain classification accuracies. LOOCV provides unbiased estimators of generalization error for stable classifiers such as KNN [33]. With LOOCV, we can also evaluate the effect of a single sample, i.e., the stability of a classifier. When feature selection is involved, the LOOCV is "external." In external LOOCV, feature selection is done *n *times separately for each set of *n *- 1 cases. The number of base classifiers for Random KNN and Random Forests is set to 2,000. The number of variables for each base classifier is set to the square-root of the total number of variables of the input dataset. Both *k *= 1 (R1NN) and *k *= 3 (R3NN) for Random KNN are evaluated.

### Performance comparison without feature selection

Random Forests and Random KNN are applied to the two groups of datasets using all genes available. The results (data not shown) indicate that Random Forests was nominally better than Random KNN on 11 datasets while Random KNN was nominally better than Random Forests on 9 datasets. They have a tie on one dataset. Using the *p*-values from the McNemar test [34], Random Forests was no better than Random KNN on any of the datasets, while R1NN was significantly better than Random Forests on the NCI data and Random Forests was better than R3NN on two datasets. Using the average accuracies, no significant difference was observed in Group I (0.80 for RF, 0.81 for R1NN, 0.78 for R3NN), or in Group II (0.86 for RF, 0.84 for R1NN, 0.86 for R3NN). Therefore from the test on the 21 datasets, we may conclude that without feature selection, Random KNN is generally equivalent to Random Forests in classification performance.

### Performance comparison with feature selection

The proposed feature selection approach using Random KNN is applied to the 21 datasets and compared with Random Forests. The proportion of features removed at each iteration was set to 0.2 for both RKNN-FS and RF-FS (since the second stage is kind of fine-tuning, to save time only stage one was used for comparison) and other parameter settings are the same as in the previous section. The results are shown in Tables 5 and 6. The indicated results are the mean, standard deviation, and coefficient of variation recorded based on the individual execution of the leave-one-out cross validation (LOOCV) procedure. In one case in the more complex datasets of Group I (**Adenocarcinoma**), RF was better than R3NN in both classification accuracy and stability, but R1NN provided a similar performance with RF in both stability and classification accuracy. In another case in Group I (**Brain**), RF was slightly better than RKNN-FS in classification accuracy, but much worse in stability of classification accuracy. In just one case in the simpler dataset of Group II (**Prostrate**), RF-FS was better than both R1NN and R3NN in both classification accuracy and stability. They had a virtual tie one one dataset (**Leukemia**). In all the other datasets (17 out of 21), RKNN-FS was better in both classification rate, and in stability of the classification rates. RKNN-FS showed much more significant performance improvements over RF on the more complex datasets of Group I. From the tables, one can observe the general trend: RKNN-FS performance improvement over RF increases with increasing dataset complexity (though not necessarily monotonically).

**Table 5:** Comparative performance with gene selection, Group I

Dataset	p * *c*/*n*	Mean Accuracy			Standard Deviation			Coefficient of Variation		
				
		RF	R1NN	R3NN	RF	R1NN	R3NN	RF	R1NN	R3NN
Ramaswamy	1267	0.577	0.726	0.704	0.019	0.013	0.013	3.231	1.775	1.796
Staunton	859	0.561	0.692	0.663	0.042	0.026	0.031	7.485	3.802	4.669
Nutt	829	0.671	0.903	0.834	0.051	0.030	0.031	7.619	3.268	3.674
Su	792	0.862	0.901	0.888	0.016	0.015	0.014	1.884	1.624	1.567
NCI	688	0.813	0.854	0.836	0.033	0.027	0.023	4.083	3.135	2.796
Brain	666	0.969	0.958	0.940	0.025	0.013	0.018	2.574	1.323	1.875
Armstrong	468	0.936	0.993	0.980	0.020	0.009	0.013	2.166	0.938	1.345
Pomeroy	329	0.858	0.933	0.863	0.025	0.016	0.017	2.892	1.762	1.991
Bhattacharjee	310	0.934	0.956	0.954	0.015	0.006	0.006	1.572	0.620	0.618
Adenocarcinoma	260	0.942	0.939	0.859	0.018	0.017	0.032	1.948	1.808	3.675
Golub	222	0.943	0.986	0.986	0.022	0.003	0.004	2.328	0.289	0.369
Singh	206	0.889	0.952	0.931	0.024	0.014	0.018	2.718	1.427	1.920

Average		0.830	0.899	0.870	0.026	0.016	0.018	3.375	1.814	2.191

**Table 6:** Comparative performance with gene selection, Group II

Dataset	p * *c*/*n*	Mean Accuracy			Standard Deviation			Coefficient of Variation		
				
		RF	R1NN	R3NN	RF	R1NN	R3NN	RF	R1NN	R3NN
Lymphoma	195	0.993	1.000	1.000	0.012	0.000	0.000	1.162	0.000	0.000
Leukemia	161	1.000	0.999	0.999	0.000	0.006	0.004	0.000	0.596	0.427
Breast.3.class	154	0.778	0.793	0.761	0.024	0.037	0.035	3.023	4.665	4.639
SRBCT	147	0.982	0.998	0.996	0.010	0.005	0.007	0.967	0.470	0.684
Shipp	142	0.865	0.997	0.991	0.033	0.008	0.011	3.757	0.800	1.077
Breast.2.class	126	0.838	0.841	0.822	0.024	0.052	0.042	2.894	6.206	5.049
Prostate	118	0.947	0.941	0.917	0.007	0.011	0.016	0.703	1.154	1.701
Khan	111	0.985	0.994	0.994	0.006	0.006	0.008	0.643	0.608	0.809
Colon	65	0.894	0.944	0.910	0.010	0.013	0.025	1.163	1.337	2.733

Average		0.920	0.945	0.932	0.014	0.015	0.016	1.590	1.760	1.902

### Stability

The tables above also show the standard deviation and coefficient of variation (multiplied by 100) for the classification accuracy of RKNN-FS and RF-FS on each dataset. The tables clearly show that RKNN-FS is much more stable with respect to classification accuracy than RF-FS. As with classification accuracy itself, the improvement in stability of the accuracy rates over RF-FS also improves with increasing complexity of the dataset. Another way to measure the stability is by considering the variability in the size of the selected gene set. At each run of the LOOCV, the size of the best gene set selected by Random KNN and Random Forests for each cross-validation is recorded. The average size and standard deviation are reported in Tables 7 and 8. From these tables, one can see that for some datasets (**NCI, Armstrong, Nutt, Pomeroy, Ramaswamy, Staunton and Su**), the standard deviation of the best gene set size could be surprisingly large with Random Forests. The standard deviation can be larger than 1000 (**Armstrong **dataset, selected feature set sizes range from 3 to 7184)! The above datasets either have more classes (≥ 4 classes) and/or a large number of genes (*p *> 10, 000), and thus have high p * *c*/*n *values. It is also believed that datasets with a lager number genes have more noisy genes than those with a smaller number of genes from which the original investigators removed some genes somehow. This shows a striking problem with Random Forests for noisy "small *n*, large *p*" datasets: the size of the selected best gene set can change dramatically even when just one data point is changed (by LOOCV). In principle, Random Forests tries to tackle the problem of instability of the tree structure by bootstrapping the data and constructing many trees. However, the above results support the fact that Random Forests is still unstable in the presence of noisy or unbalanced input. See [22,21] for further discussion on the problem of instability in Random Forests. As Table 7 shows, in general the stability of Random KNN is much better than that of Random Forests. Clearly, such a trend will be expected to have some impact on computational requirements - with the stability of RKNN-FS in the size of the selected feature sets, there will also be less variability in its computational requirements. Thus, we recommended Random KNN over Random Forests for gene selection on microarray data.

**Table 7:** Average gene set size and standard deviation, Group I

Dataset	p * *c*/*n*	Mean Feature Set Size			Standard Deviation		
			
		RF	R1NN	R3NN	RF	R1NN	R3NN
Ramaswamy	1267	907	336	275	666	34	52
Staunton	859	185	74	60	112	12	11
Nutt	829	146	49	49	85	6	4
Su	792	858	225	216	421	9	26
NCI	688	126	187	163	118	41	33
Brain	666	18	137	120	13	42	42
Armstrong	468	249	76	73	1011	16	12
Pomeroy	329	69	89	82	70	15	13
Bhattacharjee	310	33	148	146	29	15	10
Adenocarcinoma	260	8	38	11	4	20	11
Golub	222	12	27	21	8	5	5
Singh	206	26	25	13	32	6	6

Average		220	118	102	214	18	19

**Table 8:** Average gene set size and standard deviation, Group II

Dataset	p * *c*/*n*	Mean Feature Set Size			Standard Deviation		
			
		RF	R1NN	R3NN	RF	R1NN	R3NN
Lymphoma	195	75	114	103	30	49	44
Leukemia	161	2	28	36	0	22	18
Breast.3.Class	154	47	43	36	35	23	8
SRBCT	147	49	65	64	50	8	9
Shipp	142	13	46	48	23	9	6
Breast.2.Class	126	32	23	15	29	16	10
Prostate	118	16	32	15	10	10	11
Khan	111	17	67	36	5	11	14
Colon	65	21	37	36	18	5	5

Average		30	51	43	22	17	14

### Time comparison

**Figure 7:**
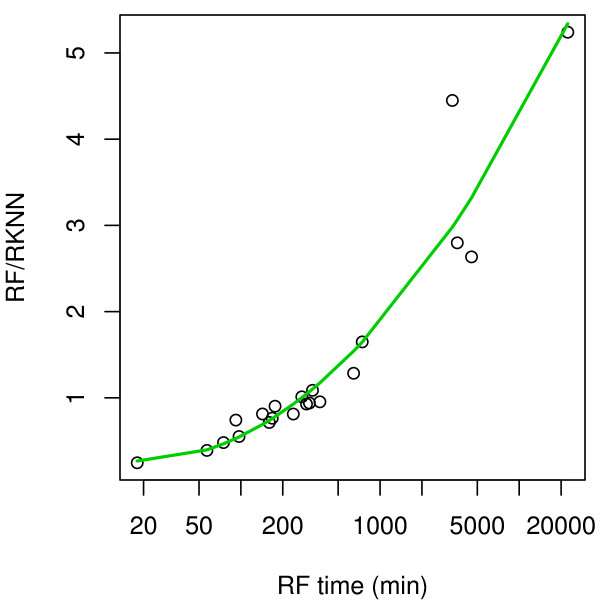
**Comparison of execution time between RKNN-FS and RF-FS**.

The computing times for RF-FS and RKNN-FS are recorded and reported in Tables 9 and 10. For the smaller (less complex) datasets (Group II), RF-FS is faster than RKNN-FS. However, as shown by time ratio in Figure 7, RKNN-FS is much faster than RF-FS on the large computationally intensive tasks. For instance, RKNN-FS is 4-5 times faster on datasets with very large *p*, and many classes (such as **Armstrong and Staunton**). We conclude that Random KNN is more *scalable *than Random Forests in feature selection. This is important, especially in dealing with the computational burden involved in very high dimensional datasets. Between R1NN and R3NN, there was little or no difference in execution time, although R1NN was slightly faster.

**Table 9:** Execution time comparison, Group I

Dataset	p * *c*/*n*	Time (min)			Ratio	
			
		RF	R1NN	R3NN	RF/R1NN	RF/R3NN
Ramaswamy	1267	22335	4262	4324	5.2	5.2
Staunton	859	3310	744	753	4.4	4.4
Nutt	829	176	195	195	0.9	0.9
Su	792	3592	1284	1279	2.8	2.8
NCI	688	142	177	178	0.8	0.8
Brain	666	92	124	125	0.7	0.7
Armstrong	468	327	301	297	1.1	1.1
Pomeroy	329	296	319	320	0.9	0.9
Bhattacharjee	310	4544	1725	1733	2.6	2.6
Adenocarcinoma	260	274	272	273	1.0	1.0
Golub	222	160	224	224	0.7	0.7
Singh	206	646	503	498	1.3	1.3

Total		35894	10130	10199	3.54	3.52

**Table 10:** Execution time comparison, Group II

Dataset	p * *c*/*n*	Time (min)			Ratio	
			
		RF	R1NN	R3NN	RF/R1NN	RF/R3NN
Lymphoma	195	57	146	147	0.4	0.4
Leukemia	161	18	74	74	0.3	0.2
Breast.3.Class	154	310	332	334	0.9	0.9
SRBCT	147	97	177	178	0.5	0.5
Shipp	142	238	293	286	0.8	0.8
Breast.2.Class	126	167	221	222	0.8	0.8
Prostate	118	370	389	391	1.0	0.9
Khan	111	745	452	451	1.6	1.7
Colon	65	75	156	157	0.5	0.5

Total		2077	2240	2240	0.93	0.93

## Conclusion

In this paper, we introduce RKNN-FS, a new feature selection method for the analysis of high-dimensional data, based on the novel Random KNN classifier. We performed an empirical study using the proposed RKNN-FS on 21 microarray datasets, and compared its performance with the popular Random Forests approach. From our comparative experimental results, we make the following observations: (1) The RKNN-FS method is competitive with the Random Forests feature selection method (and most times better) in classification performance; (2) Random Forests can be very unstable under some scenarios (e.g., noise in the input data, or unbalanced datasets), while the Random KNN approach shows much better stability, whether measured by stability in classification rate, or stability in size of selected gene set; (3) In terms of processing speed, Random KNN is much faster than Random Forests, especially on the most time-consuming tasks with large *p *and multiple classes. The concept of KNN is easier to understand than the decision tree classifier in Random Forests and is easier to implement. We have focused our analysis and comparison on Random Forests, given its popularity, and documented superiority in classification accuracy over other state-of-the-art methods [20,21]. Other results on the performance of RF and its variants are reported in [35,36]. In future work, we will perform a comprehensive comparison of the proposed RKNN-FS with these other classification and feature selection schemes, perhaps using larger and more diverse datasets, or on applications different from microarray analysis.

In summary, the RKNN-FS approach provides an effective solution to pattern analysis and modeling with high-dimensional data. In this work, supported by empirical results, we suggest the use of Random KNN as a faster and more stable alternative to Random Forests. The proposed methods have applications whenever one is faced with the "small *n*, large *p *problem", a significant challenge in the analysis of high dimensional datasets, such as in microarrays.

## Authors' contributions

SL and DAA initiated this project. SL developed the methods with the help of DAA and EJH. SL and DAA analyzed the results from the proposed methods. DAA and EJH oversaw the whole project. All authors read and approved the final manuscript.

## References

[B1] Theodoridis S Koutroumbas K Pattern recognition 2003 Academic Press

[B2] Duda RO Hart PE Stork DG Pattern Classification 2000 New York: John Wiley & Sons

[B3] Saeys Y Inza I Larrañaga P A review of feature selection techniques in bioinformatics Bioinformatics 2007 23 19 10.1093/bioinformatics/btm344 17720704

[B4] Breiman L Friedman J Stone CJ Olshen R Classification and Regression Trees 1984 Chapman & Hall/CRC

[B5] Breiman L Random Forests Machine Learning 2001 45 5 32 10.1023/A:1010933404324

[B6] Al-Ani A Deriche M Chebil J A new mutual information based measure for feature selection Intelligent Data Analysis 2003 7 43 57

[B7] Last M Kandel A Maimon O Information-theoretic algorithm for feature selection Pattern Recognition Letters 2001 22 6-7 799 811 10.1016/S0167-8655(01)00019-8

[B8] Song GJ Tang SW Yang DQ Wang TJ A Spatial Feature Selection Method Based on Maximum Entropy Theory Journal of Software 2003 14 9 1544 1550

[B9] Mitra P Murthy C Pal S Unsupervised feature selection using feature similarity Pattern Analysis and Machine Intelligence, IEEE Transactions on 2002 24 3 301 312 10.1109/34.990133

[B10] Kira K Rendell LA A practical approach to feature selection ML92: Proceedings of the ninth international workshop on Machine learning 1992 San Francisco, CA: Morgan Kaufmann Publishers Inc 249 256

[B11] Kononenko I Šimec E Robnik-Šikonja M Overcoming the Myopia of Inductive Learning Algorithms with RELIEFF Applied Intelligence 1997 7 39 55 10.1023/A:1008280620621

[B12] Hall MA Smith LA Feature Selection for Machine Learning: Comparing a Correlation-based Filter Approach to the Wrapper Proceedings of the Twelfth Florida International Artificial Intelligence Research Symposium Conference 1999 Menlo Park, CA: The AAAI Press 235 239

[B13] Whitley D Ford M Livingstone D Unsupervised Forward Selection: A Method for Eliminating Redundant Variables Journal of Chemical Information and Computer Science 2000 40 5 1160 1168 10.1021/ci000384c 11045809

[B14] Yu L Liu H Efficient Feature Selection via Analysis of Relevance and Redundancy The Journal of Machine Learning Research 2004 5 1205 1224

[B15] Kohavi R John GH Wrappers for feature selection Artificial Intelligence 1997 97 1-2 273 324 10.1016/S0004-3702(97)00043-X

[B16] Blum A Langley P Selection of relevant features and examples in machine learning Artificial Intelligence 1997 97 1-2 245 271 10.1016/S0004-3702(97)00063-5

[B17] Akaike H A new look at the statistical model identification IEEE Transactions on Automatic Control 1974 19 6 716 723 10.1109/TAC.1974.1100705

[B18] Schwarz G Estimating the dimension of a model The Annals of Statistics 1978 6 2 461 4643 10.1214/aos/1176344136

[B19] Zhai HL Chen XG Hu ZD A new approach for the identification of important variables Chemometrics and Intelligent Laboratory Systems 2006 80 130 135 10.1016/j.chemolab.2005.09.002

[B20] Li S Fedorowicz A Singh H Soderholm SC Application of the Random Forest Method in Studies of Local Lymph Node Assay Based Skin Sensitization Data Journal of Chemical Information and Modeling 2005 45 4 952 964 10.1021/ci050049u 16045289

[B21] Díaz-Uriarte R de Andrés SA Gene selection and classification of microarray data using random forest BMC Bioinformatics 2006 7 3 10.1186/1471-2105-7-3 PMC1363357 16398926

[B22] Strobl C Boulesteix AL Zeileis A Hothorn T Bias in random forest variable importance measures: Illustrations, sources and a solution BMC Bioinformatics 2007 8 25 10.1186/1471-2105-8-25 PMC1796903 17254353

[B23] Calle ML Urrea V Letter to the Editor: Stability of Random Forest importance measures Briefings in Bioinformatics 2011 12 86 89 10.1093/bib/bbq011 20360022

[B24] Nicodemus KK Letter to the Editor: On the stability and ranking of predictors from random forest variable importance measures Briefings in Bioinformatics 12 4 369 373 10.1093/bib/bbr016 PMC3137934 21498552

[B25] Li S Random KNN Modeling and Variable Selection for High Dimensional Data PhD thesis 2009 West Virginia University

[B26] Fix E Hodges J Discriminatory Analysis-Nonparametric Discrimination: Consistency Properties 1951 Tech. Rep. 21-49-004, 4, US Air Force, School of Avaiation Medicine

[B27] Cover T Hart P Nearest Nieghbor Pattern Classification IEEE Transaction on Information Theory 1967 IT-13 21 27

[B28] Hastie T Tibshirani R Friedman J The Elements of Statistical Learning - Data Mining, Inference, and Prediction 2001 New York: Springer chap. 9, section 2

[B29] Crookston NL Finley AO yaImpute: An R Package for kNN Imputation Journal of Statistical Software 2007 23 10 1 16

[B30] Troyanskaya O Cantor M Sherlock G Brown P Hastie T Tibshirani R Botstein D Altman RB Missing value estimation methods for DNA microarrays Bioinformatics 2001 17 6 520 525 10.1093/bioinformatics/17.6.520 11395428

[B31] Ho TK The Random Subspace Method for Constructing Decision Forests IEEE Transactions on Pattern Analysis and Machine Intelligence 1998 20 8 832 844 10.1109/34.709601

[B32] Dietterich TG Machine-Learning Research: Four Current Directions The AI Magazine 1998 18 4 97 136

[B33] Breiman L Heuristics of instability and stabilization in model selection The Annals of Statistics 1996 24 6 2350 2383

[B34] McNemar Q Note on the sampling error of the difference between correlated proportions or percentages Psychometrika 1947 12 153 157 10.1007/BF02295996 20254758

[B35] Svetnik V Liaw A Tong C Culberson J Sheridan R Feuston B Random Forest: A Classification and Regression Tool for Compound Classification and QSAR Modeling Journal of Chemical Information and Computer Science 2003 43 6 1947 1958 10.1021/ci034160g 14632445

[B36] Lin Y Jeon Y Random Forests and Adaptive Nearest Neighbors Journal of the American Statistical Association 2006 101 474 578 590 10.1198/016214505000001230

